# Neglected Mycoses in Brazil: A Population‐Based Study of Mortality and In‐Hospital Mortality Over 25 Years

**DOI:** 10.1111/myc.70144

**Published:** 2026-02-11

**Authors:** Anderson Fuentes Ferreira, Jorg Heukelbach, Eliana Amorim de Souza, Maria Aparecida Shikanai‐Yasuda, Lisandra Serra Damasceno, Fernanda Dockhorn Costa, Terezinha do Menino Jesus Silva Leitão, Helia Kawa, Alberto Novaes Ramos

**Affiliations:** ^1^ Programa de Pós‐Graduação Em Saúde Pública, Faculdade de Medicina Universidade Federal Do Ceará Fortaleza Ceará Brazil; ^2^ Instituto Multidisciplinar de Saúde, Universidade Federal da Bahia, Campus Anísio Teixeira Vitória da Conquista Bahia Brazil; ^3^ Departamento de Infectologia e Medicina Tropical, Faculdade de Medicina Universidade de São Paulo São Paulo Brazil; ^4^ Departamento de Saúde Comunitária, Faculdade de Medicina Universidade Federal Do Ceará Fortaleza Ceará Brazil; ^5^ Departamento de HIV/Aids, Tuberculose, Hepatite Viral e ISTs, Secretaria de Vigilância Em Saúde e Ambiente Ministério da Saúde Brasília Brazil; ^6^ Departamento de Epidemiologia e Bioestatística Universidade Federal Fluminense Rio de Janeiro Brazil

**Keywords:** hospitalisation, in‐hospital mortality, mortality, mycoses, neglected tropical diseases, spatiotemporal analysis

## Abstract

**Objective:**

To describe the epidemiology, associated factors, spatial distribution, and temporal trends of mortality and in‐hospital mortality related to systemic mycoses in Brazil, 2000–2024.

**Methods:**

This is a nationwide ecological study combining temporal and spatial analyses using death certificates (DC; underlying and/or associated causes) and hospital admissions (HA; primary and/or secondary diagnoses) with in‐hospital deaths. We estimated rate ratios (RR) with 95% confidence intervals (CI) interpreted as comparative mortality and in‐hospital mortality rates between sociodemographic categories at the aggregate level, stratified by sex and age group; temporal trends were presented with the average annual percent change (AAPC) and 95% CIs; spatial heterogeneity was described across states. Outcomes were expressed as population‐based rates (per 100,000 inhabitants).

**Results:**

We identified 22,230 mycosis‐related deaths among a total of 30,488,786 deaths (0.07%), corresponding to an overall mortality rate of 0.46 per 100,000 population. Mortality was higher in males (RR 2.91; 95% CI 2.51–3.38) and peaked at ages 60–69 years (RR 3.47; 95% CI 2.67–4.51). Nationwide mortality declined over time (AAPC −1.12; 95% CI −1.41 to −0.83). Deaths were geographically concentrated in the states of Rondônia, Mato Grosso, Goiás and Mato Grosso do Sul. We recorded a total of 4471 in‐hospital deaths among 11,367,369 admissions (0.04%), yielding an in‐hospital mortality rate of 0.09 per 100,000 population. In‐hospital risk of death was higher in males (RR 2.12; 95% CI 1.55–2.89) and in those aged ≥ 70 years (RR 12.50; 95% CI 7.38–21.17). No significant nationwide trend was observed for in‐hospital mortality (AAPC 0.64; 95% CI −1.20 to 2.59). Spatial distribution during the analysis period was heterogeneous, especially in the states of Rondônia, São Paulo, Rio de Janeiro, Paraíba and Paraná.

**Conclusion:**

Our findings fill an important gap by jointly analysing long‐term mortality and in‐hospital mortality related to systemic mycoses at a nationwide scale, using two complementary information systems. Neglected mycoses remain an important cause of death in Brazil, including deaths during hospitalisation. Distinct individual‐level and spatial patterns support the need for strengthened surveillance, prevention, control, and therapeutic strategies within the Brazilian Unified Health System.

## 1 Introduction

Symptoms caused by fungal infections range from asymptomatic or mild presentations, including superficial infections, to severe pneumonia and disseminated disease involving multiple organs. Infections are particularly consequential in immunosuppressed hosts, such as solid‐organ transplant recipients [1] and people living with HIV [2, 3]. Clinical manifestations may vary depending on the host's immune status and can lead to skin lesions and severe systemic disease [1]. Clinical management can be challenging, often requiring prolonged therapy and, in selected cases, surgical intervention [4, 5].

In the context of the World Health Organization (WHO) classification of neglected tropical diseases (NTDs), mycetoma, chromoblastomycosis and other deep mycoses are grouped under a single NTD category in the WHO Road map for neglected tropical diseases 2021–2030 [6]. NTDs also encompass conditions caused by bacteria, helminths, protozoa and other parasites, viruses, food‐borne trematodiases and snakebite envenoming [6]. Paracoccidioidomycosis, although clinically and epidemiologically important in Latin America, is not currently listed as an NTD, but its aetiological agents (*Paracoccidioides* spp.) are classified as ‘medium priority’ pathogens in the WHO Fungal Priority Pathogens List (FPPL) [7]. This distinction between NTDs and priority fungal pathogens is relevant for surveillance, funding and policy advocacy and is explicitly considered in our analyses.

As with other NTDs, fungal diseases remain neglected because, as evidenced by resource constraints and knowledge gaps in surveillance, diagnostics and treatment [6, 8]. These infections can be stigmatising and contribute to cycles of inequality and poverty; effective prevention and control require approaches that address environmental determinants and community well‐being [8, 9, 10, 11, 12].

The impact of fungal diseases may be exacerbated by climate variability and change, particularly in settings with wide fluctuations in humidity and temperature [13, 14]. For instance, paracoccidioidomycosis, human infection with *Paracoccidioides* spp., typically follows soil disturbance, including large‐scale earth removal, and disproportionately often affects rural workers, with substantial economic consequences [12, 15, 16, 17].

Fungal infections are more prevalent in tropical and subtropical regions, with a substantial burden in Latin America, as well as in parts of Africa and Asia, with differing at‐risk populations; at the same time, several invasive fungal diseases such as candidaemia and aspergillosis have global relevance and cause high mortality in intensive care settings worldwide. Overall, many of the fungal pathogens considered in this study are distributed globally, although their burden is shaped by regional environmental and social determinants [2, 6, 8, 18, 19, 20].

Cryptococcosis is distributed worldwide and remains a common opportunistic illness where HIV prevalence is high and health services are limited [6, 21, 22]. Reflecting public health priority, the 2022 WHO Fungal Priority Pathogens list classifies *Cryptococcus neoformans* as a ‘critical priority’, *Histoplasma* spp. and eumycetoma agents as ‘high priority’, and *Paracoccidioides* spp., *Coccidioides* spp., and *Cryptococcus gattii* as ‘medium priority’ [7].

In the global agenda, the Sustainable Development Goals (SDGs) include these conditions under Goal 3 (‘Ensure healthy lives and promote well‐being for all at all ages’), with Target 3.3 calling for the end of the epidemics of AIDS, tuberculosis, malaria, and NTDs by 2030 [8]. Progress was hindered by the COVID‐19 pandemic, which disrupted community interventions, timely diagnosis and treatment and supply chains, while diverting attention and resources away from NTD programmes [23].

In Brazil, with the exception of human sporotrichosis, a nationally notifiable disease since March 2025, most systemic mycoses are not subject to national notification and lack routine epidemiologic surveillance, although some states have adopted local reporting measures (e.g., Rio de Janeiro, Rio Grande do Sul, Mato Grosso do Sul, Paraná, and São Paulo States) [24]. Several of these states are important agricultural producers and have populations exposed to land‐use change, deforestation and soil disturbance associated with the expansion of plantations and pastures, which may increase the risk of endemic mycoses. Workers who become ill are removed from work and enter the social protection system, generating demand for Brazilian social protection systems. These diseases are of concern in specific settings, with cases in humans and animals and notable impacts in urban centres and smaller states [25, 26]. They account for important fractions of infectious‐disease mortality [27], occur in localised outbreaks [16, 19, 28] and carry high fatality in immunosuppressed populations [21, 22]—with approximately 30% case fatality rates reported for histoplasmosis [3] and ~20% for cryptococcosis [22]. Recent federal measures included the incorporation of new antifungal treatments (voriconazole, isavuconazole and anidulafungin) into the public system, and the national notification of human sporotrichosis; key challenges include workforce training and establishing a diagnostic‐treatment network with rapid testing and antifungal availability [24, 29].

Given the absence of robust case estimates, Brazil's nationwide information systems provide a valuable information source on disease burden, particularly the Mortality Information System (*Sistema de Informação sobre Mortalidade*, SIM) and the Hospital Information System of the Unified Health System (*Sistema de Informações Hospitalares do SUS*, SIH‐SUS) [30, 31, 32]. Leveraging these large, long‐standing datasets enables representative analyses to inform priority‐setting and stimulate research on neglected mycoses at the national level [31, 32]. To the best of our knowledge, no previous study has analysed long‐term national trends in both mortality and in‐hospital mortality for a broad group of systemic and neglected mycoses in Brazil, using SIM and SIH‐SUS in an integrated way.

Accordingly, this study presents mortality and in‐hospital mortality related to neglected fungal infections in Brazil, 2000–2024, describing national, regional, state‐level, and municipal patterns and temporal trends, and exploring their association with sociodemographic and contextual characteristics.

## 2 Methods

### 2.1 Study Design

This nationwide, population‐based ecological study analysed deaths related to neglected mycoses in Brazil. We analysed secondary data from the Mortality Information System (SIM) based on death certificates (DC; underlying and/or associated causes) and from the Hospital Information System (SIH‐SUS), using variables from the Hospital Admission Authorization (AIH; primary and/or secondary diagnoses) [30, 31]. All analyses were conducted at aggregate levels (country, macro‐region, state and municipality), and information on age and sex was used for rate standardisation and stratification only; no individual‐level inferences were made, and potential ecological fallacy is explicitly acknowledged. We included the COVID‐19 pandemic period to assess potential public health impacts of reduced coverage within the SUS care network and resulting changes in patterns and trends [23].

### 2.2 Study Area

**FIGURE 1:**
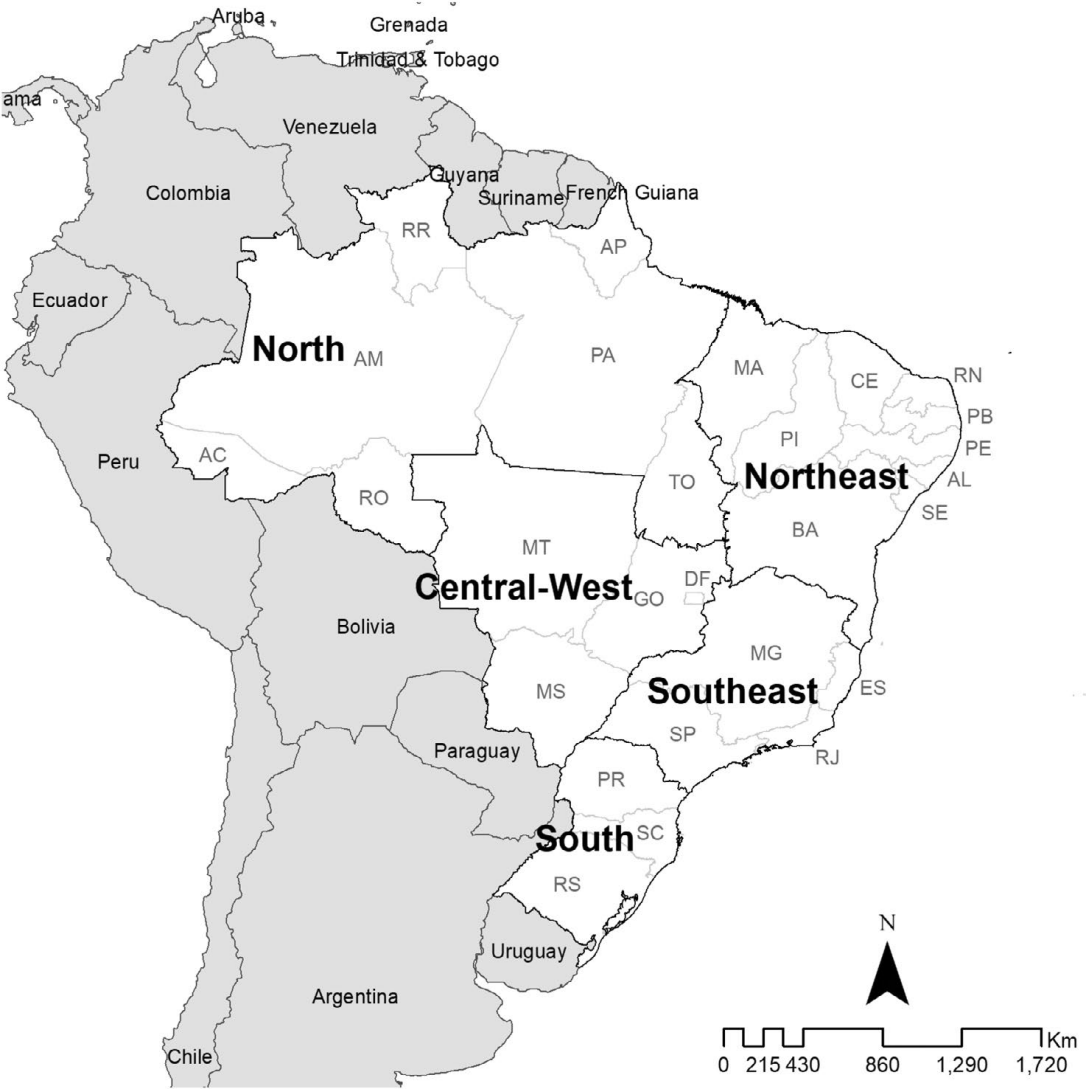
Study area, Brazil: Macro regions and states. North (AC: Acre, AP: Amapá, AM: Amazonas, PA: Pará, RO: Rondônia, RR: Roraima, and TO: Tocantins), Northeast (AL: Alagoas, BA: Bahia, CE: Ceará, MA: Maranhão, PB: Paraíba, PE: Pernambuco, PI: Piauí, RN: Rio Grande do Norte, and SE: Sergipe), Southeast (ES: Espírito Santo, MG: Minas Gerais, RJ: Rio de Janeiro, and SP: São Paulo), South (PR: Paraná, RS: Rio Grande do Sul, and SC: Santa Catarina), and Central‐West (DF: Federal District, GO: Goiás, MT: Mato Grosso, and MS: Mato Grosso do Sul).

Brazil is the fifth‐largest country by land area and the largest in South America by population, covering 8.5 million km^2^ and an estimated 212 million inhabitants in 2024 (population density 23.86 inhabitants/km^2^). Administratively, it comprises 5570 municipalities, 26 states, the Federal District and five macro regions (North, Northeast, Southeast, South and Central‐West) (Figure 1).

Marked economic, environmental, social and cultural diversity coexists with heterogeneous public health service organisations. The least populous macro regions are the Central‐West (8.02% of the population) and the North (8.54%) [33]; together with the Northeast, they include municipalities with the lowest social indicators [33, 34]. According to the 2022 Brazilian Census, 45% of the population self‐identified as Mixed/Pardo and 44% as White, with 8.3% of households lacking waste collection, 16.1% lacking a main water supply, and 35.3% not connected to a sewerage system [34].

### 2.3 Data Sources

We obtained DC and AIH data for 2000–2024 from the Ministry of Health (MoH) virtual environment (DATASUS), specifically the SIM and SIH‐SUS databases. Both systems contain individual‐level records with sociodemographic and clinical variables and are decentralised nationwide.

SIM was created in 1975 and digitised in the late 1970s; subsequent decentralisation followed SUS implementation [30, 35]. The SIH‐SUS was established in 1991 to register hospital admission events in SUS‐affiliated and public hospitals (approximately 75% of national admissions), initially for administrative and financial purposes; the AIH is the core registration instrument [31].

Causes were classified using the International Statistical Classification of Diseases and Related Health Problems, 10th revision (ICD‐10) [36]. We selected ICD‐10 codes for NTD‐listed fungal infections: chromoblastomycosis/chromomycosis (B43), mycetoma (B47), paracoccidioidomycosis (B40, B41) and sporotrichosis (B42) [6]. In 1971, the Pan American Health Organization standardised the term ‘South American blastomycosis’ (ICD‐10 B40) as paracoccidioidomycosis [37]. We also included coccidioidomycosis (B38), cryptococcosis (B45) and histoplasmosis (B39) given their clinical–epidemiologic relevance in Brazil [4, 9, 13, 36, 38, 39]. For HIV co‐infection, we assessed B20–B24 (HIV disease codes). In both SIM and SIH‐SUS systems, we adopted the multiple‐causes‐of‐disease approach, considering selected mycoses when recorded as underlying or associated causes of death (SIM) or as primary or secondary diagnoses (SIH‐SUS). A given DC or AIH could contain more than one ICD‐10 code of interest. However, each death or hospitalisation was counted only once in the analyses, even when multiple relevant codes were present.

Datasets were downloaded in .dbc format by year and state and converted to .dbf using TabWin 4.15. Data management and statistical analyses were performed in Stata 11.2 (StataCorp, College Station, TX, USA).

Population denominators were obtained from the Brazilian Institute of Geography and Statistics (IBGE) via DATASUS, using census counts (2000, 2010, 2022) and intercensal projections (2001–2009, 2011–2021, 2023–2024) by municipality, including age and sex. We used mid‐year population estimates for rate calculations and, where indicated, applied direct standardisation by age and sex using the Brazilian population as reference to enhance comparability across periods and regions.

We excluded non‐hospitalised cases and admissions in private hospitals not contracted by SUS. Given the neglected profile of most included mycoses, we assumed that the majority of relevant HA were captured in SIH‐SUS—given that approximately 75% of hospital admissions in Brazil are recorded in this system [29]. Thus, our hospital analyses primarily reflect admissions financed by SUS, and we acknowledge that a residual fraction of admissions occurring exclusively in the private sector may not be captured.

### 2.4 Variables

Variables included cause of hospitalisation (primary; secondary), underlying cause of death, associated causes of death, place of death (hospital; other facility; home; public street), HIV co‐infection (yes; no), sex (female; male), age group in years (0–14; 15–29; 30–39; 40–49; 50–59; 60–69; ≥ 70), self‐reported ethnicity (White; Black/Afro‐descendant; Asian‐descendant; Mixed/Pardo; Indigenous), region of residence (North; Northeast; Southeast; South; Central‐West), municipality size (small I; small II; medium; large), state capital residence (no; yes), the Brazilian Index of Deprivation (*Índice Brasileiro de Privação*, IBP) (very low; low; medium; high; very high) [40, 41] and the IBGE municipal typology (urban; intermediate adjacent; intermediate remote; rural adjacent; rural remote)—used as a proxy for urban–rural gradients, population density and accessibility to health services, particularly for in‐hospital mortality analyses [42]. The IBP summarises material deprivation (schooling, income and household infrastructure), and municipal typology summarises infrastructure and size, both calculated from 2010 census data [40, 41].

Municipality size was defined as: small I (≤ 20,000 inhabitants), small II (20,001–50,000), medium (50,001–100,000), and large (> 100,000), based on data from the 2010 census. The IBGE typology supports territorial planning by combining population thresholds with the distribution across densely populated areas [42]. Ethnicity population data were based on the 2010 census. Because these contextual indicators (IBP, municipality size and typology, and ethnicity distributions) derive from the 2010 census, they were treated as time‐invariant characteristics in our analyses and used to describe differential patterns in recent periods and spatial distributions rather than to estimate year‐by‐year changes.

### 2.5 Data Analysis

We computed absolute and relative frequencies (including missing values)—aggregating data at national level and across the entire period analysed. Multiple admissions for the same person may occur and could not be distinguished; thus, the unit of analysis for SIH‐SUS was the admission record. For mortality, SIM records each death as a unique event, so duplicate death records are not expected.

Municipalities of residence (*N* = 5570) were the spatial unit—used for data aggregation in the periods analysed; records with unknown or invalid municipality codes were excluded from spatial analyses but retained in national and regional summaries when other key variables were valid. Spatial classes for municipal indicators (per 100,000 inhabitants) were defined using Jenks natural breaks—a classification method that minimises within‐class variance and maximises between‐class variance, thereby enhancing the identification of meaningful spatial clusters and reducing the undue influence of extreme values compared with equal‐interval or quantile‐based categories.

We calculated mortality rates per 100,000 inhabitants as the number of mycosis‐related deaths divided by the resident population (population average of the periods). We also calculated in‐hospital mortality rates per 100,000 inhabitants as the number of in‐hospital deaths due to selected mycoses divided by the resident population (population average of the periods). When indicated, in‐hospital case‐fatality was computed as deaths among admissions with selected mycoses divided by such admissions, expressed as a percentage.

For time‐series displays, we used age‐ and sex‐standardised rates (direct method) for Brazil and macro‐regions, with annual mortality and in‐hospital mortality. Rate ratios (RR) with 95% confidence intervals (CI) were estimated to compare aggregate rates between sociodemographic categories; these RRs should be interpreted as comparative measures between groups in an ecological framework and not as individual‐level causal ‘relative risks’. Pearson's χ^2^ tested significance of differences—considering the average number of deaths or hospital mortality for the period in relation to the population in each category, based on data from the 2010 census.

Temporal trends were assessed using joinpoint regression (Joinpoint Regression Program, version 5.2.0.0). The best‐fit segmented model was selected via the Monte Carlo permutation method—the model indicated by the analysis was selected as the model that best represents the segment. The maximum number of joinpoints allowed in each model followed the default recommendations of the software according to the length of the time series, and a 5% significance level was adopted for testing changes in trend. We report annual percent change (APC) and average annual percent change (AAPC) with 95% CIs; trends were categorised as decreasing (negative APC/AAPC, statistically significant), increasing (positive APC/AAPC, statistically significant) or showing no defined trend (not statistically significant).

Spatial analyses used age‐sex standardised rates (direct method) by municipalities weighted to the 2010 Brazil age‐sex structure (per 100,000 inhabitants) for 2000–2004, 2005–2009, 2010–2014, 2015–2019, and 2020–2024 (population average of the periods).

### 2.6 Ethical Considerations

Data were obtained anonymised from official, publicly accessible MoH sources (SIM: ftp://ftp.datasus.gov.br/dissemin/publicos/SIHSUS/, ftp://ftp.datasus.gov.br/dissemin/publicos/SIM/CID10/DORES/; SIH‐SUS: ftp://ftp.datasus.gov.br/dissemin/publicos/SIHSUS/) via the DATASUS/MoH portal, with no personal identifiers. The study complies with National Health Council Resolution No. 466/2012 on research involving human subjects and with Brazil's General Law on Personal Data Protection (Law No. 13,709).

## 3 Results

### 3.1 Mortality

Over the 25‐year period, a total of 30,488,786 deaths were recorded nationwide, of which 22,230 (0.07%) were related to fungal infections. Cryptococcosis accounted for approximately two‐thirds of these deaths, followed by paracoccidioidomycosis and histoplasmosis; other mycoses were less frequent (Table 1).

**TABLE 1:** Bivariate analysis of factors associated with mycosis‐related mortality and in‐hospital mortality, Brazil, 2000–2024.

Indicator/Variables	Mortality				Hospital mortality			
	*N* (%)	Crude rate (per 100,000 inhabitants)	Standardised rate (per 100,000 inhabitants) (95% CI)	RR (95% CI)	*N* (%)	Crude rate (per 100,000 inhabitants)	Standardised rate (per 100,000 inhabitants) (95% CI)	RR (95% CI)
Brazil – Total	**22,230 (100.0)**	**0.46**	**0.45 (0.45–0.46)**	—	**4471 (100.0)**	**0.09**	**0.09 (0.09–0.09)**	—
Chromoblastomycosis/Chromomycosis	64 (0.3)	0.00	0.00 (0.00–0.00)	—	31 (0.7)	0.00	0.00 (0.00–0.00)	—
Coccidioidomycosis	139 (0.6)	0.00	0.00 (0.00–0.00)	—	1,193 (26.7)	0.02	0.02 (0.02–0.03)	—
Cryptococcosis	13,354 (60.1)	0.27	0.27 (0.27–0.28)	—	1,686 (37.7)	0.03	0.03 (0.03–0.04)	—
Histoplasmosis	3,518 (15.8)	0.07	0.07 (0.07–0.07)	—	297 (6.6)	0.01	0.01 (0.01–0.01)	—
Mycetoma	119 (0.5)	0.00	0.00 (0.00–0.00)	—	7 (0.2)	0.00	0.00 (0.00–0.00)	—
Paracoccidioidomycosis	4904 (22.1)	0.10	0.10 (0.10–0.10)	—	1,136 (25.4)	0.02	0.02 (0.02–0.02)	—
Sporotrichosis	205 (0.9)	0.00	0.00 (0.00–0.00)	—	123 (2.8)	0.00	0.00 (0.00–0.00)	—
Cause of hospital admission								
Primary	—	—	—	—	3,623 (81.0)	—	—	—
Secondary	—	—	—	—	848 (19.0)	—	—	—
Cause of death								
Underlying	6,197 (27.9)	—	—	—	—	—	—	—
Associated	16,033 (72.1)	—	—	—	—	—	—	—
Place where death occurred								
Hospital	21,092 (94.9)	—	—	—	—	—	—	—
Other	429 (1.9)	—	—	—	—	—	—	—
Home	654 (2.9)	—	—	—	—	—	—	—
Public roads	45 (0.2)	—	—	—	—	—	—	—
Missing data	65 (0.3)	—	—	—	—	—	—	—
HIV co‐infection								
Yes	12,482 (56.1)	—	—	—	520 (11.6)	—	—	—
No	9,748 (43.9)	—	—	—	3,951 (88.4)	—	—	—
Sex								
Female	5,848 (26.3)	0.24	0.23 (0.23–0.24)	Reference	1,483 (33.2)	0.06	0.06 (0.06–0.06)	Reference
Male	16,380 (73.7)	0.69	0.68 (0.67–0.69)	2.91 (2.51–3.38)	2988 (66.8)	0.13	0.12 (0.12–0.13)	2.12 (1.55–2.89)
Missing data	2 (0.0)	—	—	—	—	—	—	—
Age group								
0–14	238 (1.1)	0.02	0.02 (0.02–0.02)	0.08 (0.04–0.16)	187 (4.2)	0.02	0.02 (0.01–0.02)	0.38 (0.16–0.90)
15–29	3,223 (14.5)	0.25	0.26 (0.25–0.26)	Reference	507 (11.3)	0.04	0.04 (0.04–0.04)	Reference
30–39	5,519 (24.8)	0.74	0.72 (0.7–0.74)	2.97 (2.39–3.69)	613 (13.7)	0.08	0.08 (0.07–0.09)	2.17 (1.20–3.90)
40–49	5,127 (23.1)	0.81	0.8 (0.78–0.82)	3.28 (2.63–4.09)	707 (15.8)	0.11	0.11 (0.1–0.12)	2.89 (1.63–5.14)
50–59	3,774 (17)	0.81	0.77 (0.75–0.8)	3.26 (2.58–4.13)	696 (15.6)	0.15	0.14 (0.13–0.15)	3.90 (2.20–6.93)
60–69	2,486 (11.2)	0.86	0.77 (0.74–0.8)	3.47 (2.67–4.51)	640 (14.3)	0.22	0.2 (0.18–0.22)	5.88 (3.28–10.53)
≥ 70	1,852 (8.3)	0.79	0.71 (0.68–0.75)	3.19 (2.39–4.24)	1,121 (25.1)	0.48	0.43 (0.41–0.46)	12.5 (7.38–21.17)
Missing data	(0)	—	—	—	—	—	—	—
Ethnicity*								
Caucasian	11,374 (51.2)	0.50	—	Reference	1,261 (28.2)	0.06	—	Reference
Afro‐Brazilian/Afro‐descendant	2225 (10.0)	0.62	—	1.24 (0.98–1.55)	187 (4.2)	0.05	—	0.88 (0.40–1.95)
Asian‐descendant	86 (0.4)	0.16	—	0.28 (0.09–0.88)	49 (1.1)	0.09	—	1.72 (0.42–7.08)
Mixed/Pardo Brazilians	7,408 (33.3)	0.36	—	0.71 (0.61–0.82)	972 (21.7)	0.05	—	0.85 (0.56–1.30)
Indigenous (Amerindians)	85 (0.4)	0.41	—	0.73 (0.23–2.26)	5 (0.1)	0.02	—	1.09 (0.07–17.70)
Missing data	1,052 (4.7)	—	—		1,997 (44.7)	—	—	—
Region of residence								
North	2073 (9.3)	0.51	0.57 (0.55–0.60)	1.07 (0.84–1.35)	457 (10.2)	0.11	0.14 (0.12–0.15)	1.29 (0.70–2.38)
Northeast	2667 (12.0)	0.20	0.21 (0.20–0.21)	0.41 (0.33–0.51)	1137 (25.4)	0.08	0.09 (0.08–0.09)	0.97 (0.59–1.58)
Southeast	9,819 (44.2)	0.48	0.45 (0.44–0.46)	Reference	1,964 (43.9)	0.10	0.09 (0.09–0.09)	1.12 (0.71–1.77)
South	5,055 (22.7)	0.72	0.68 (0.66–0.70)	1.51 (1.28–1.79)	594 (13.3)	0.09	0.08 (0.07–0.09)	Reference
Central‐West	2616 (11.8)	0.73	0.72 (0.69–0.74)	1.53 (1.23–1.9)	319 (7.1)	0.09	0.09 (0.08–0.10)	1.05 (0.54–2.07)
Size of municipality								
Small I	2763 (12.4)	0.35	0.34 (0.33–0.36)	0.87 (0.66–1.15)	744 (16.6)	0.09	0.09 (0.08–0.1)	0.98 (0.65–1.47)
Small II	2773 (12.5)	0.35	0.36 (0.34–0.37)	0.87 (0.66–1.15)	605 (13.5)	0.08	0.08 (0.07–0.08)	0.78 (0.50–1.21)
Medium	2327 (10.5)	0.40	0.41 (0.39–0.43)	Reference	460 (10.3)	0.08	0.08 (0.07–0.09)	0.80 (0.49–1.32)
Large	14,337 (64.5)	0.53	0.51 (0.51–0.52)	1.3 (1.05–1.62)	2662 (59.5)	0.10	0.10 (0.09–0.1)	Reference
Missing data	30 (0.1)	—	—	—	—	—	—	—
Residence in the capital								
No	15,881 (71.4)	0.43	0.42 (0.42–0.43)	Reference	3,482 (77.9)	0.09	0.09 (0.09–0.10)	1.09 (0.77–1.55)
Yes	6,349 (28.6)	0.55	0.53 (0.52–0.55)	1.28 (1.1–1.48)	989 (22.1)	0.09	0.08 (0.08–0.09)	Reference
IBP								
Very low	6,073 (27.3)	0.68	0.63 (0.61–0.64)	1.30 (1.08–1.57)	964 (21.6)	0.11	0.10 (0.09–0.11)	Reference
Low	4809 (21.6)	0.54	0.52 (0.50–0.53)	1.03 (0.85–1.26)	765 (17.1)	0.09	0.08 (0.08–0.09)	0.79 (0.50–1.27)
Medium	5,090 (22.9)	0.52	0.51 (0.49–0.52)	Reference	841 (18.8)	0.09	0.08 (0.08–0.09)	0.80 (0.50–1.26)
High	4067 (18.3)	0.42	0.42 (0.41–0.43)	0.80 (0.65–0.99)	1,122 (25.1)	0.12	0.12 (0.11–0.13)	1.06 (0.69–1.63)
Very high	2157 (9.7)	0.19	0.21 (0.20–0.21)	0.37 (0.28–0.47)	779 (17.4)	0.07	0.07 (0.07–0.08)	0.63 (0.39–1.01)
Missing data	34 (0.2)	—	—	—	—	—	—	—
Typology of municipality								
Urban	18,805 (84.6)	0.51	0.50 (0.49–0.51)	1.58 (1.15–2.16)	3,583 (80.1)	0.10	0.10 (0.09–0.10)	Reference
Intermediate adjacent	1,028 (4.6)	0.32	0.32 (0.30–0.34)	Reference	259 (5.8)	0.08	0.08 (0.07–0.09)	0.81 (0.43–1.54)
Intermediate remote	146 (0.7)	0.44	0.51 (0.42–0.59)	1.39 (0.59–3.28)	29 (0.6)	0.09	0.10 (0.06–0.13)	0.77 (0.11–5.53)
Rural adjacent	1,912 (8.6)	0.26	0.26 (0.25–0.27)	0.81 (0.55–1.18)	540 (12.1)	0.07	0.07 (0.07–0.08)	0.78 (0.50–1.22)
Rural remote	305 (1.4)	0.34	0.38 (0.34–0.43)	1.03 (0.54–1.97)	60 (1.3)	0.07	0.07 (0.06–0.09)	0.57 (0.14–2.32)
Missing data	34 (0.2)	—	—	—	—	—	—	—

Abbreviations: %, Percentage; −: not calculated; IBP: Brazilian Index of Deprivation (*Índice Brasileiro de Privação*); N: Number; RR, Relative Risk.

* ethnicity data available from 2008 for hospitalisations.

**FIGURE 2:**
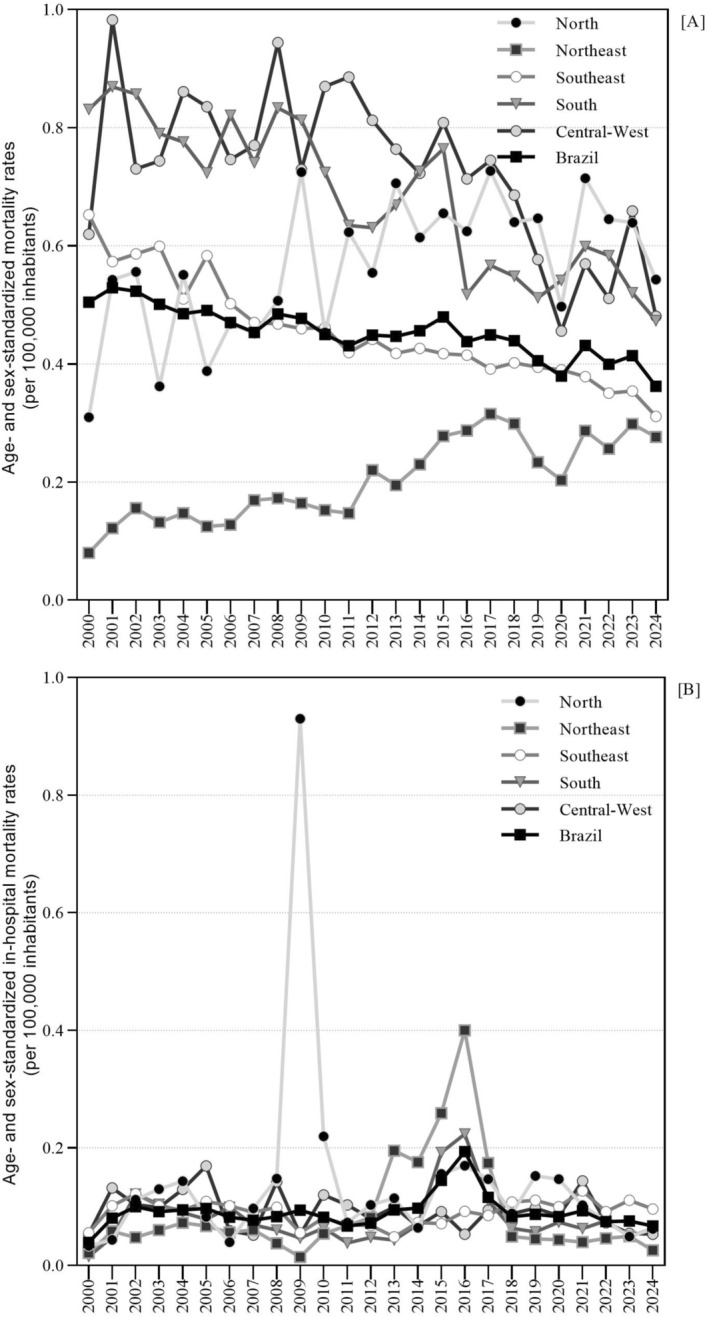
Age‐ and sex‐standardised mycosis‐related rates (per 100,000 inhabitants) by region and country, Brazil, 2000–2024: (A) mortality; (B) in‐hospital mortality.

The mean mortality rate was 0.45 per 100,000 inhabitants, with adjustments made for age and sex. While the national mean rate declined over time, adjusted rates increased in the Northeast (0.21 per 100,000; 95% CI 0.20–0.21) and North (0.57 per 100,000; 95% CI 0.55–0.60) (Figure 2A; Table 1).

Approximately three‐quarters of mycosis‐related deaths were certified as associated causes, and one‐quarter as underlying causes (Table 1). Nearly all deaths occurred in hospital settings. Most deaths from systemic mycoses occurred among individuals with HIV co‐infection, aged 30–49 years, self‐identified as Caucasians (‘white’), residing in the Southeast region, in non‐capital urban municipalities, and in areas with high deprivation (Table 1). HIV co‐infection was most frequent among deaths related to histoplasmosis (76.5%, 2692/3,518) and cryptococcosis (71.4%, 9,534/13,354).

Higher rates were observed in males; in those aged 60–69 years; in the afro‐descendant group; in residents of the South; in large municipalities and state capitals; in areas classified with very low IBP; and in municipalities with an urban typology (Table 1). These RRs represent comparative mortality rates between categories rather than individual‐level risks.

The overall temporal trend in mortality was downward, similarly for males and females. Among mixed/pardo Brazilians, a temporary increasing trend was observed during 2000–2017. Regionally, rates increased in the North and Northeast, whereas other regions declined. Upward trends were also seen in municipalities with very high IBP and in rural remote municipalities (Table 2).

**TABLE 2:** Temporal trends in crude mycosis‐related mortality and in‐hospital mortality rates (per 100,000 inhabitants) by sociodemographic strata, Joinpoint regression, Brazil, 2000–2024.

Indicator/Variables	Mortality			Hospital mortality		
	Period	APC (95% CI)	AAPC (95% CI)	Period	APC (95% CI)	AAPC (95% CI)
Brazil – Total	2000–2024	−1.12* (−1.41;–0.83)	−1.12* (−1.41;–0.83)	2000–2012	−0.64 (−14.87;3.29)	0.64 (−1.20;2.59)
				2012–2016	17.58* (2.26;35.82)	
				2016–2024	−11.19* (−19.04;–6.75)	
Sex						
Female	2000–2024	−0.63* (−1.15;‐0.12)	−0.63* (−1.15;–0.12)	2000–2012	2.11 (−17.80;8.62)	1.15 (−1.50;4.03)
				2012–2016	19.28 (−25.64;39.30)	
				2016–2024	−16.38* (−27.52;–0.76)	
Male	2000–2024	−1.28* (−1.61;–0.97)	−1.28* (−1.61;–0.97)	2000–2012	−1.94 (−14.29;1.31)	0.49 (−1.18;2.25)
				2012–2016	16.84* (3.42;34.23)	
				2016–2024	−8.55* (−16.22;–4.65)	
Age group						
0–14	2000–2024	−0.23 (−2.34;1.71)	−0.23 (−2.34;1.71)	2000–2004	31.99 (−3.00;257.85)	−10.03* (−15.62;–7.92)
				2004–2024	−13.13* (−23.77;–11.24)	
15–29	2000–2005	−7.63* (−19.28;–2.39)	−1.00* (−1.98;–0.06)	2000–2005	10.45* (3.11;23.16)	−1.35 (−3.52;0.65)
	2005–2024	0.14 (−0.75;2.37)		2005–2009	−27.37* (−38.37;–17.56)	
				2009–2024	6.63* (4.48;9.61)	
30–39	2000–2024	−3.68* (−4.18;–3.23)	−3.68* (−4.18;–3.23)	2000–2024	−2.85* (−5.49;‐0.43)	−2.85* (−5.49;–0.43)
40–49	2000–2002	12.12 (−2.19;27.19)	−2.15* (−2.76;–1.55)	2000–2024	−0.66 (−2.12;0.90)	−0.66 (−2.12;0.90)
	2002–2024	−2.47* (−7.37;–1.84)				
50–59	2000–2024	−1.42* (−2.20;–0.55)	−1.42* (−2.20;–0.55)	2000–2024	−1.11 (−2.58;0.48)	−1.11 (−2.58;0.48)
60–69	2000–2024	−1.45* (−2.14;–0.71)	−1.45* (−2.14;–0.71)	2000–2016	4.98* (1.88;52.96)	0.69 (−1.89;4.36)
				2016–2024	−8.38* (−30.80;–0.72)	
≥ 70	2000–2024	−1.97* (−2.82;–1.07)	−1.97* (−2.82;–1.07)	2000–2016	15.45* (11.90;24.92)	1.57 (−3.60;9.22)
				2016–2024	−28.28* (−41.19;‐21.90)	
Ethnicity*						
Caucasian	2000–2024	−1.80* (−2.08;–1.53)	−1.80* (−2.08;–1.53)	−	−	−
Afro‐Brazilian/Afro‐descendant	2000–2024	−2.07* (−2.77;–1.31)	−2.07* (−2.77;–1.31)	−	−	−
Asian‐descendant	2000–2010	−21.04* (−54.23;–10.08)	−1.06 (−5.86;3.53)	−	−	−
	2010–2024	16.59* (5.85;45.52)				
Mixed/Pardo Brazilians	2000–2017	2.25* (1.51;18.98)	1.43* (0.86;2.08)	−	−	−
	2017–2024	−1.09 (−12.96;1.27)				
Indigenous (Amerindians)	2000–2024	1.04 (−1.74;5.23)	1.04 (−1.74;5.23)	−	−	−
Region of residence						
North	2000–2024	1.96* (1.02;3.10)	1.96* (1.02;3.10)	2000–2009	33.48 (−25.08;205.12)	−3.62 (−9.50;1.95)
				2009–2012	−39.38 (−56.68;109.67)	
				2012–2024	−0.70 (−16.94;51.85)	
Northeast	2000–2017	6.12* (4.79;17.62)	4.32* (3.20;5.77)	2000–2009	−2.41 (−43.13;17.49)	4.60 (−1.15;12.75)
	2017–2024	−0.51 (−12.66;3.55)		2009–2016	30.62 (−47.08;85.87)	
				2016–2024	−30.61 (−44.94;3.12)	
Southeast	2000–2008	−3.72* (−8.00;‐2.56)	−2.51* (−2.87;–2.16)	2000–2002	35.35 (−2.70;86.19)	0.43 (−0.97;1.95)
	2008–2024	−2.00 (−2.53;0.69)		2002–2012	−5.22 (−20.43;9.00)	
				2012–2024	4.60 (−3.25;12.92)	
South	2000–2024	−2.35* (−2.96;‐1.78)	−2.35* (−2.96;–1.78)	2000–2012	−6.89* (−18.53;‐1.17)	0.48 (−2.71;3.96)
				2012–2015	58.93* (15.60;94.17)	
				2015–2024	−14.83* (−23.78;–10.19)	
Central‐West	2000–2011	0.84 (−1.20;10.11)	−1.79* (−2.77;–0.82)	2000–2024	−1.60 (−3.90;0.63)	−1.60 (−3.90;0.63)
	2011–2024	−3.83* (−9.97;–2.29)				
Municipality size						
Small I	2000–2024	0.23 (−0.35;0.84)	0.23 (−0.35;0.84)	2000–2011	0.11 (−27.37;11.48)	1.85 (−1.65;5.88)
				2011–2015	29.39 (−34.16;64.52)	
				2015–2024	−15.20 (−26.12;16.03)	
Small II	2000–2024	−0.12 (−0.80;0.59)	−0.12 (−0.80;0.59)	2000–2016	4.46* (1.64;45.19)	0.82 (−1.55;3.37)
				2016–2024	−9.08* (−35.24;‐1.00)	
Medium	2000–2024	−1.60* (−2.44;–0.79)	−1.60* (−2.44;–0.79)	2000–2002	131.44* (24.73;363.83)	−2.49 (−5.33;0.20)
				2002–2010	−10.22* (−29.08;–3.86)	
				2010–2024	0.69 (−3.46;24.18)	
Large	2000–2024	−1.53* (−2.00;–1.04)	−1.53* (−2.00;–1.04)	2000–2013	0.27 (−15.86;4.67)	0.81 (−1.08;2.84)
				2013–2016	21.07 (−9.61;34.33)	
				2016–2024	−10.28* (−20.05;–2.37)	
Residence in the capital						
No	2000–2024	−1.02* (−1.32;–0.72)	−1.02* (−1.32;–0.72)	2000–2012	−1.40 (−17.02;3.48)	0.92 (−1.53;3.63)
				2012–2016	23.76* (5.61;48.96)	
				2016–2024	−13.49* (−22.21;–8.45)	
Yes	2000–2024	−1.35* (−1.87;–0.84)	−1.35* (−1.87;–0.84)	2000–2024	−0.71 (−3.06;1.65)	−0.71 (−3.06;1.65)
IBP						
Very low	2000–2024	−3.20* (−3.54;–2.90)	−3.20* (−3.54;–2.90)	2000–2013	−4.50 (−13.38;2.25)	0.55 (−0.79;2.04)
				2013–2016	24.23 (−5.86;38.02)	
				2016–2024	−2.72 (−14.63;2.97)	
Low	2000–2024	−2.85* (−3.38;–2.35)	−2.85* (−3.38;‐2.35)	2000–2024	−0.02 (−1.55;1.60)	−0.02 (−1.55;1.60)
Medium	2000–2024	−0.52* (−0.93;‐0.11)	−0.52* (−0.93;‐0.11)	2000–2024	0.31 (−1.42;2.23)	0.31 (−1.42;2.23)
High	2000–2024	0.66 (−0.02;1.42)	0.66 (−0.02;1.42)	2000–2016	9.27* (5.17;19.39)	1.27 (−2.83;6.21)
				2016–2024	−19.99* (−41.82;–11.65)	
Very high	2000–2017	5.36* (4.30;7.91)	3.70* (2.82;4.79)	2000–2011	−1.23 (−24.16;8.19)	1.83 (−2.70;7.10)
	2017–2024	−0.91 (−8.54;2.21)		2011–2016	28.86* (6.17;68.84)	
				2016–2024	−26.47* (−38.23;–19.53)	
Typology of municipality						
Urban	2000–2024	−1.52* (−1.81;–1.23)	−1.52* (−1.81;–1.23)	2000–2013	−0.41 (−15.62;4.11)	0.49 (−1.16;2.28)
				2013–2016	20.39 (−9.80;33.29)	
				2016–2024	−9.68* (−18.78;–2.55)	
Intermediate adjacent	2000–2018	1.83* (0.89;8.39)	0.84 (−0.06;1.81)	2000–2013	−0.62 (−40.24;242.82)	1.47 (−1.87;5.29)
	2018–2024	−3.98 (−18.67;0.33)		2013–2016	39.45 (−47.67;82.35)	
				2016–2024	−16.07 (−38.00;47.78)	
Intermediate remote	2000–2024	−0.18 (−2.67;2.74)	−0.18 (−2.67;2.74)	2000–2020	6.33* (2.89;19.49)	3.02 (−1.29;8.89)
				2020–2024	−42.09* (−82.79;–5.84)	
Rural adjacent	2000–2024	0.66 (−0.19;1.55)	0.66 (−0.19;1.55)	2000–2011	−1.46 (−24.16;7.37)	1.38 (−1.94;4.99)
				2011–2015	33.20 (−14.44;71.42)	
				2015–2024	−18.00* (−27.59;–11.21)	
Rural remote	2000–2024	2.20* (0.76;3.93)	2.20* (0.76;3.93)	2000–2024	−2.33 (−6.31;1.37)	−2.33 (−6.31;1.37)

Abbreviations: −: not calculated; AAPC: average annual percent change; APC: annual percent change; CI: 95% confidence intervals; IBP: Brazilian Index of Deprivation (Índice Brasileiro de Privação).

* Significantly different from 0 (*p* < 0.05); 95%.

* ethnicity data available from 2008 for hospitalisations.

**FIGURE 3:**
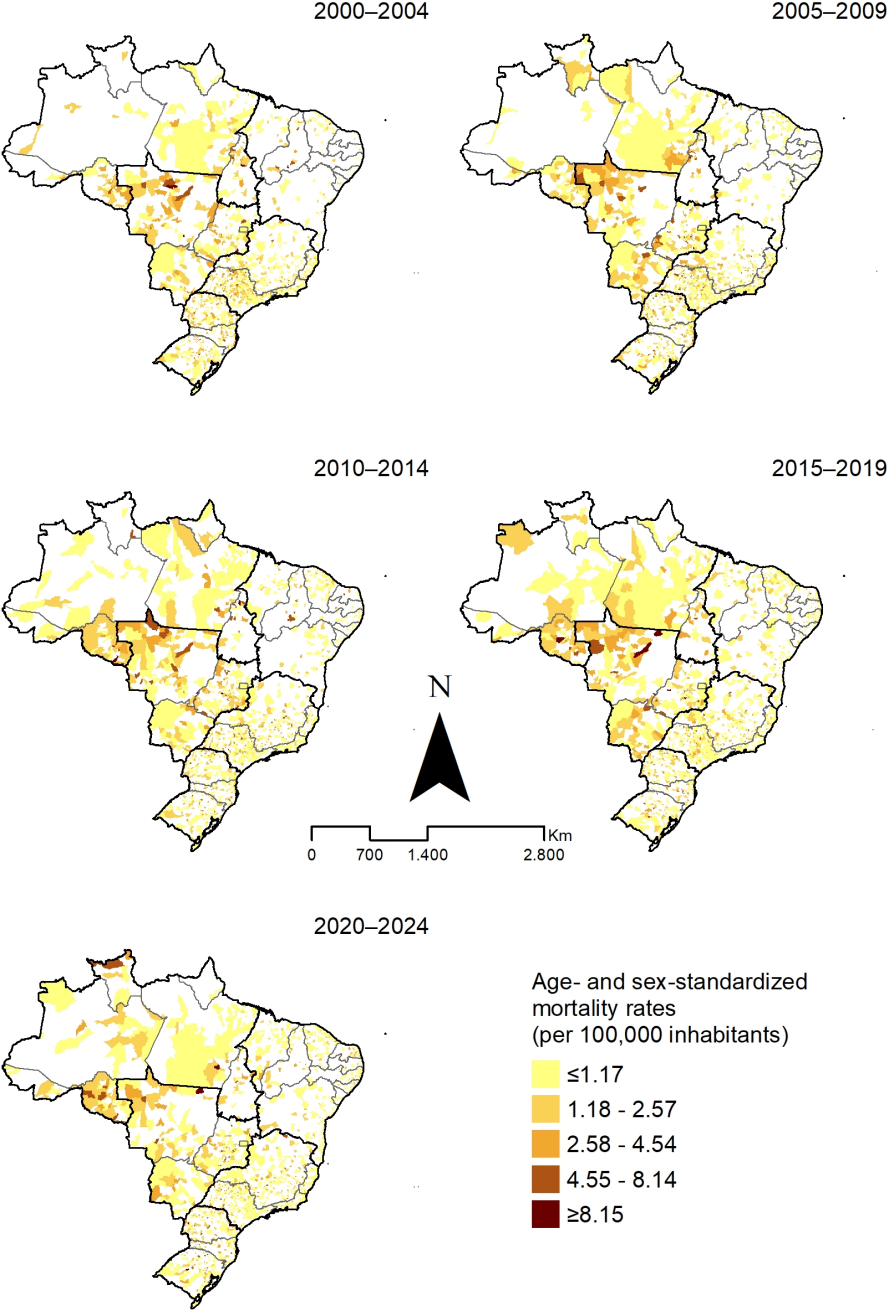
Spatial distribution of age‐ and sex‐standardised mycosis‐related mortality rates (per 100,000 inhabitants), Brazil, 2000–2024.

Spatially, adjusted mortality rates displayed a consistent pattern of higher concentrations in northern areas of Mato Grosso, Rondônia (particularly after 2010), Goiás and Mato Grosso do Sul. Other states showed heterogeneous patterns, generally with rates ≤ 1.17 per 100,000 inhabitants (Figure 3).

### 3.2 In‐Hospital Mortality

Across the same period, 290,543,535 hospital admissions (HAs) were recorded, of which 11,367,369 (3.9%) resulted in in‐hospital death. Among these in‐hospital deaths, 4471 (0.04%) were attributed to fungal infections, predominantly cryptococcosis and coccidioidomycosis (Table 1). No clear national or regional temporal trend in the in‐hospital mortality rate was observed. A peak was observed for the Northern region in 2009 (Figure 2B).

In most HAs, a mycosis was recorded as the primary cause of hospitalisation. Patients who died in hospital were predominantly male, aged ≥ 70 years, self‐identified as Caucasians, and not HIV co‐infected (Table 1). In‐hospital deaths were concentrated in the Southeast, in urban areas, in municipalities with > 100,000 inhabitants, in non‐capital municipalities, and in areas with high deprivation. Risk was highest among men and older adults (Table 1).

Of the deaths that occurred in hospital, 11.6% (520/4471) were due to HIV co‐infection. The most frequent causes of death were histoplasmosis (31.6%, 94/297) and cryptococcosis (24.1%, 406/1,686). Proportionally, the mortality rate was higher among hospital admissions involving HIV co‐infection (20.1%, or 520 out of 2586 cases) than among deaths without a recorded HIV co‐infection (8.8%, or 3951 out of 44,790 cases) (Tables 1, S1 and S3).

Over the entire period, no significant temporal overall trend in the in‐hospital mortality rate was detected, except among those aged 0–14 and 30–39 years; an increasing segment was observed from 2012 to 2016 (Table 2). The 15–29‐year age group showed increases during 2000–2005 and 2009–2024. The South region exhibited an increase in 2012–2015. Municipalities with 20,001–50,000 and 50,001–100,000 inhabitants showed increases in 2000–2016 and 2000–2002, respectively. Municipalities with High and Very high IBP increased in 2000–2016 and 2011–2016, respectively. Intermediate remote municipalities increased in 2000–2020 (Table 2).

**FIGURE 4:**
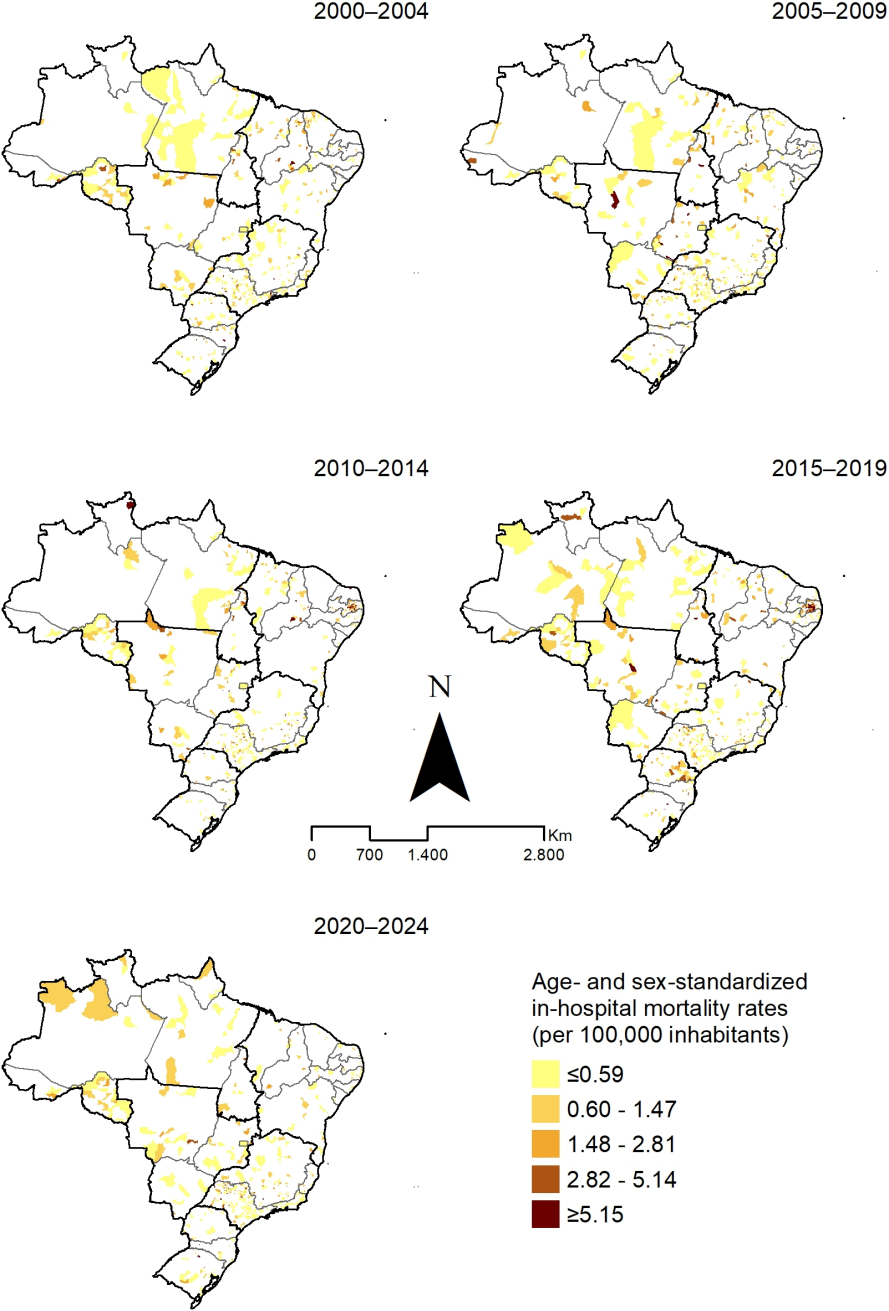
Spatial distribution of age‐ and sex‐standardised in‐hospital mortality rates for mycoses (per 100,000 inhabitants), Brazil, 2000–2024.

Spatially, adjusted in‐hospital mortality rates were heterogeneous, with higher rates (≥ 5.15 per 100,000 inhabitants) concentrated in selected municipalities within Rondônia (except 2005–2009), São Paulo, Rio de Janeiro, eastern Paraíba (2010–2019) and eastern Paraná (2015–2019). Elsewhere, rates were ≤ 1.48 per 100,000 inhabitants, with no consistent clustering by area or period (Figure 4).

## 4 Discussion

In this nationwide analysis, deaths related to fungal infections represented a meaningful public health burden in Brazil, with disease‐related mortality persisting over 25 years. The countrywide presence of these infections helps to explain the sustained levels of hospitalisations and overall mycosis‐related mortality observed [2, 43]. Overall, population‐based mortality rates declined nationwide, whereas no clear trend emerged for in‐hospital mortality. The latter pattern may reflect under‐registration and differential access to health services across settings, compounded by disruptions during the COVID‐19 period [23, 31, 32]. Events were more frequent in larger municipalities, and rates were higher among males, mixed/pardo Brazilians, and older adults.

Spatial analyses identified high‐risk areas for mortality and in‐hospital mortality, especially in remote regions historically affected by other neglected tropical diseases (NTDs) [44]. We also found a higher proportion of deaths and in‐hospital mortality among men, with distinct age profiles between SIM and SIH‐SUS records—patterns consistent with prior reports on mycoses in Brazil [22, 27, 37, 38, 45]. These findings underscore that fungal NTDs remain concentrated in specific age strata, while other NTDs such as Chagas disease often lead to death at more advanced ages because of their chronic course [44]. The spatial clusters observed in some states and municipalities are likely shaped by a combination of environmental factors (e.g., deforestation, agricultural expansion, and soil disturbance), social vulnerability, and the uneven distribution of diagnostic and referral services.

Although Caucasian ethnicity was proportionally more frequent in SIM records, the highest mortality rates occurred among afro‐descendant Brazilians. Ethnicity data were unavailable in earlier years, limiting temporal comparisons [30, 46, 47]. In Brazil, socially vulnerable groups, often disadvantaged in education and income, face precarious living conditions and barriers to care. Our findings align with evidence linking fungal diseases to social vulnerability and structural determinants [9, 13, 41, 48, 49], and argue for integrated strategies that couple fungal‐specific actions with broader NTD policies [8, 44].

We observed higher rates of deaths and in‐hospital mortality in larger municipalities, which typically have better diagnostic and treatment capacities, an indication of better access to the health system, especially for severe cases referred from smaller or remote areas [48]. Reductions in population‐based mortality likely indicate system improvements and social gains captured by census‐based indicators [34] and area‐level deprivation metrics [49], whereas the relative stability of in‐hospital mortality signals persistent access and care‐quality constraints [48, 49]. Recent policy advances, including incorporation of new antifungals into the public system and national notification of human sporotrichosis, are important steps; sustaining impact will require workforce training and a diagnostic‐treatment network with timely access to tests and medicines [24, 29].

Even amid overall improvements, people in vulnerable areas remain at risk because of overlapping drivers (e.g., HIV‐related immunosuppression and environmental exposures) [7, 22]. The COVID‐19 pandemic likely contributed to interruptions in prevention, diagnosis, and continuity of care [23], and case series have described adverse interactions between COVID‐19 and endemic mycoses [50]. Notably, we did not detect increasing mortality or in‐hospital mortality among males over time despite their higher baseline risk—these sex disparities were also observed across other NTDs [29, 44]. Elevated rates among males and adults aged 15–59 years plausibly reflect occupational and environmental exposures (e.g., soil disturbance) and immunosuppression [22, 37, 38, 45].

Trends showing declining mortality in the economically active population coupled with pockets of increased in‐hospital mortality among young adults mirror patterns described previously for mycoses in Brazil [13, 22, 27, 49], even though this age group is not the principal focus of many NTD agendas [8, 44]. Between 2007 and 2024, Brazil recorded over 540,000 HIV infections [51], which heightens susceptibility to opportunistic fungal infections and fatal outcomes [7]. Working‐age adults may also experience greater exposure to environmental sources of infection [43, 52]. In our study, HIV co‐infections accounted for more than 70% of deaths related to cryptococcosis and histoplasmosis, underscoring the strong relationship between the HIV/AIDS response in Brazil and the burden of systemic mycoses. These deaths tend to concentrate among socially marginalised groups, such as people experiencing homelessness, transgender individuals and commercial sex workers, who face multiple barriers to timely diagnosis, linkage to care and continuity of antiretroviral therapy [7, 22, 51]. A socioepidemiological perspective on ‘who continues to die of HIV/AIDS’ in Brazil is therefore essential to understand and address the persistence of mycosis‐related mortality.

In municipalities with higher deprivation indices, mortality increased, and in‐hospital mortality rose in those with medium deprivation, signals of persistent inequalities in income, education, and living conditions. The situation is widening the gap between isolated and better connected municipalities [34, 48, 49]. The North and Northeast, regions with the most adverse sociodemographic indicators, showed increasing mortality in line with prior evidence on both fungal infections and broader NTD patterns [22, 44]. Despite regional heterogeneity, deaths and in‐hospital mortality from fungal infections were documented in all states, consistent with the wide geographic distribution of pathogenic fungi [43]. In 2009, Manaus, the capital of the Brazilian state of Amazonas, saw an increase in hospital mortality attributable to fungal infections. This can be attributed to the direct influence of climatic factors [13].

Mortalities tended to increase in municipalities with ≤ 50,000 inhabitants and to decrease in state capitals, broadly echoing patterns seen for other NTDs; in‐hospital mortality rose in medium‐sized municipalities, highlighting inequities in service networks across different urban–rural typologies [44, 48]. These disparities likely stem from combined effects of service availability, referral pathways, and social and environmental conditions [34, 48, 49].

The progressive use of diagnostic tests within primary health care (PHC), access to antifungal therapy, early detection of HIV and initiation of antiretroviral therapy (ART), and improved intensive care management can contribute to reducing these trends [3, 7, 17, 19, 27, 39]. At the same time, our findings on HIV co‐infection indicate that advances in ART coverage and testing have not fully translated into equitable prevention of severe fungal disease and death, particularly in marginalised populations and regions with weaker health‐system capacity, driven by changes in surveillance policies and health inequalities affecting marginalised groups, such as homeless people, transgender individuals, and sex workers [51].

From a policy perspective, the sizable exposed population and the mortality/hospital burden support integrating fungal‐disease control into existing NTD platforms with national visibility (e.g., Chagas disease, leprosy, and leishmaniasis), aligning with the SDG 3.3 targets and Brazil's NTD agendas [8, 44]. Strengthening surveillance (including routine reporting beyond sporotrichosis), expanding laboratory capacity, and ensuring antifungal availability are pragmatic priorities [24, 29, 44]. In this context, progressively incorporating systemic mycoses into the national notifiable diseases information system (Sistema de Informação de Agravos de Notificação, SINAN), and reinforcing occupational health surveillance for rural and other at‐risk workers would increase the visibility of these conditions and support more equitable access to diagnosis and treatment.

Possible factors associated with areas with high rates can be explained by differences in diagnostic capacity, referral networks, laboratory infrastructure, accessibility to health services, or local environmental and occupational conditions [13, 14, 19, 22, 25, 33, 37, 38].

While the use of SIM and SIH‐SUS provides near‐universal, long‐run coverage and standardised coding, enabling robust national and subnational assessments, our study is subject to limitations, such as regional variability in performance of data collection, incomplete capture of private sector admissions, and misclassification or under‐registration of causes, despite documented improvements and ongoing qualification efforts [30, 31, 47]. SIH‐SUS data may suffer from under‐reporting and variable completion of diagnostic fields [29]. Another limitation concerns the impossibility of linking the SIM and SIH‐SUS databases. These limitations may influence mortality and in‐hospital mortality records. We cannot follow individual patients across hospitalisations and subsequent deaths, nor verify diagnostic concordance between systems. We mitigated biases by using underlying and associated causes in SIM and primary and secondary diagnoses in SIH‐SUS (with expanded variables after 2015), but deterministic linkage between systems was not possible. Taken together, the study limitations likely result in conservative (underestimated) burden estimates and reinforce the need to strengthen diagnostic capacity, coding practices and data integration for systemic mycoses in Brazil.

## 5 Conclusions

By jointly analysing SIM and SIH‐SUS over a 25‐year period, our study provides a comprehensive picture of mortality and in‐hospital mortality patterns that can inform national and subnational planning, including the prioritisation of fungal diagnostics, antifungal drug procurement, and targeted interventions in high‐burden areas.

Endemic mycoses are important yet neglected causes of mortality and in‐hospital mortality in Brazil. Our integrated, population‐based analysis underscores a sustained burden with pronounced individual‐ and spatial‐level heterogeneity. These findings call for inclusion of selected mycoses into the nationwide notifiable disease lists, and for strengthening laboratory capacity, surveillance, prevention, and treatment, particularly for socially and geographically vulnerable populations. Advancing equity within the SUS will require improved access to timely diagnosis, antifungal therapy, and referral pathways across regions, age, and sex groups.

Drawing on large, population‐based datasets that span nearly a quarter century, our study offers policy‐relevant evidence to support decision‐making and to prioritise surveillance, prevention, and care for neglected mycoses within SUS, particularly for populations facing social vulnerability and geographic isolation [8, 44, 48, 49].

## Funding

The authors have nothing to report.

## Conflicts of Interest

The authors declare no conflicts of interest.

## Supporting information


**Figure S1:** Age‐ and sex‐standardised mycosis‐related hospitalisation rates (per 100,000 inhabitants) by region, Brazil, 2000–2024.

**Figure S2:** Spatial distribution of age‐ and sex‐standardised hospitalisation rates for mycoses (per 100,000 inhabitants), Brazil, 2000–2024.

**Table S1:** Bivariate analysis of factors associated with hospitalisations due to mycoses, Brazil, 2000–2024.

**Table S2:** Temporal trends in crude hospitalisation rates for mycoses (per 100,000 inhabitants) by sociodemographic strata, Joinpoint regression, Brazil, 2000–2024.

**Table S3:** HIV co‐infection in mycosis‐related: Mortality, Hospitalisations and Hospital mortality, Brazil, 2000–2024.

## Data Availability

The data that support the findings of this study are available in Brazilian Ministry of Health (DATASUS) at https://datasus.saude.gov.br/servicos/. These data were derived from the following resources available in the public domain: ‐ Mortality Information System (SIM), https://ftp.datasus.gov.br/dissemin/publicos/SIM/CID10/DORES/‐ Hospital Information System (SIH‐SUS), ftp://ftp.datasus.gov.br/dissemin/publicos/SIHSUS/.
