# Supplementary material for: Neglected Mycoses in Brazil: A Population‐Based Study of Mortality and In‐Hospital Mortality Over 25 Years
Source: Mycoses. 2026 Feb 11;69(2):e70144. doi: 10.1111/myc.70144 (PMC12892236; doi:10.1111/myc.70144)

**Supplementary material - Figure 1:** Age- and sex-standardized mycosis-related hospitalization rates (per 100,000 inhabitants) by region, Brazil, 2000–2024.


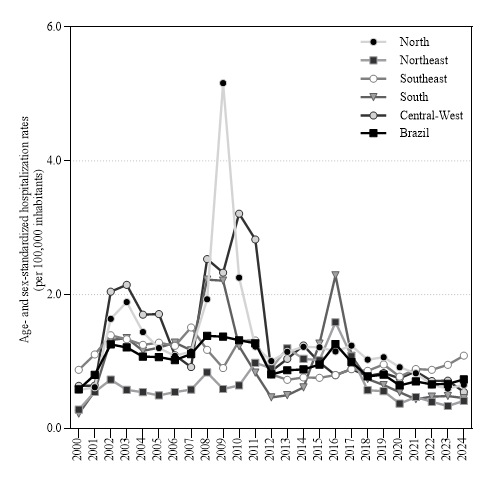

Supplement: Supplementary file 1 — Figure S1: Age‐ and sex‐standardised mycosis‐related hospitalisation rates (per 100,000 inhabitants) by region, Brazil, 2000–2024. [file MYC-69-e70144-s004.docx]
