# Supplementary material for: Neglected Mycoses in Brazil: A Population‐Based Study of Mortality and In‐Hospital Mortality Over 25 Years
Source: Mycoses. 2026 Feb 11;69(2):e70144. doi: 10.1111/myc.70144 (PMC12892236; doi:10.1111/myc.70144)

**Supplementary material - Figure 2:** Spatial distribution of age- and sex-standardized hospitalization rates for mycoses (per 100,000 inhabitants), Brazil, 2000–2024.


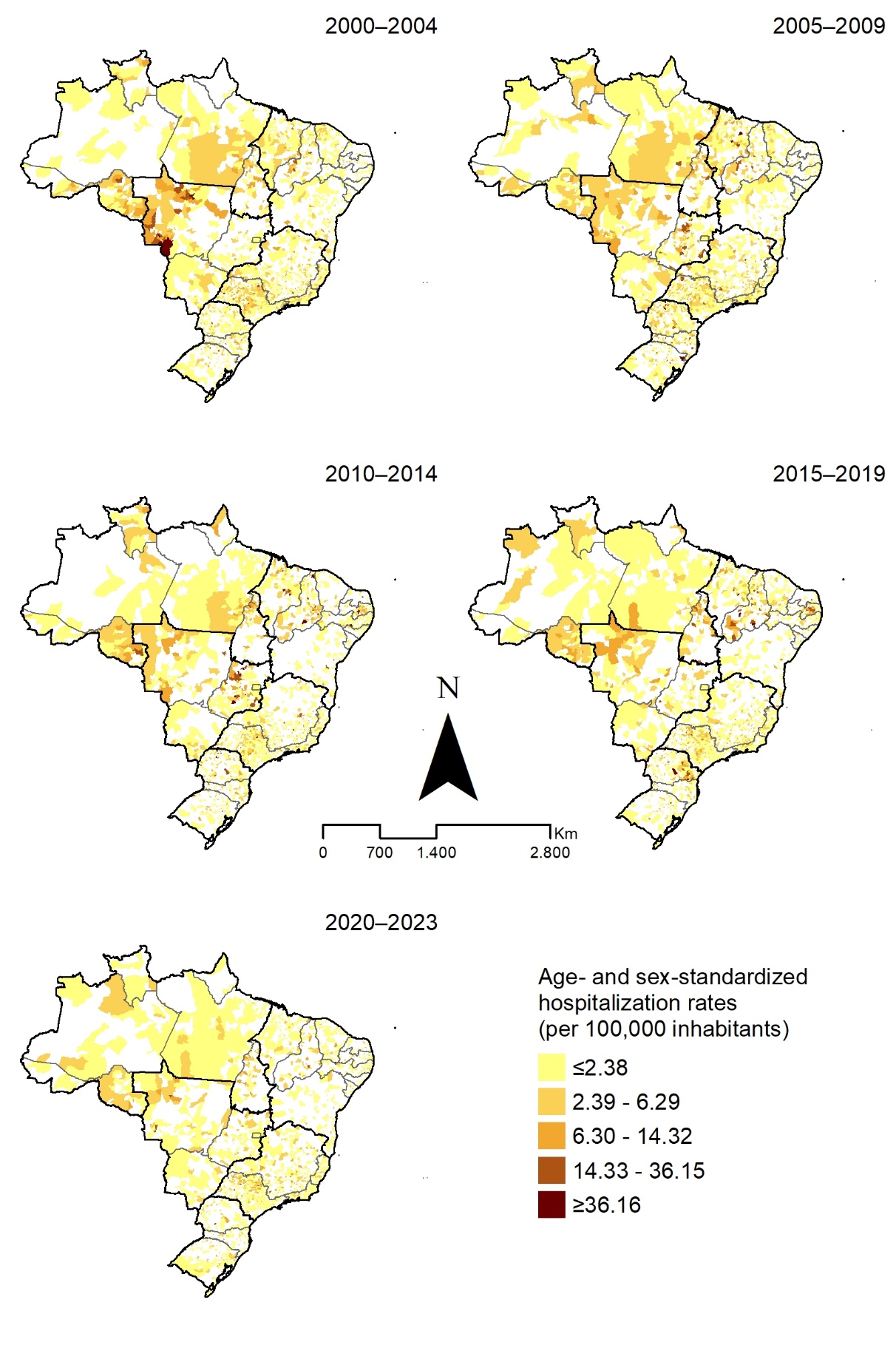

Supplement: Supplementary file 2 — Figure S2: Spatial distribution of age‐ and sex‐standardised hospitalisation rates for mycoses (per 100,000 inhabitants), Brazil, 2000–2024. [file MYC-69-e70144-s001.docx]
