# Supplementary material for: Neglected Mycoses in Brazil: A Population‐Based Study of Mortality and In‐Hospital Mortality Over 25 Years
Source: Mycoses. 2026 Feb 11;69(2):e70144. doi: 10.1111/myc.70144 (PMC12892236; doi:10.1111/myc.70144)
Supplement: Supplementary file 3 — Table S1: Bivariate analysis of factors associated with hospitalisations due to mycoses, Brazil, 2000–2024. [file MYC-69-e70144-s003.docx]

**Supplementary material - Table 1:** Bivariate analysis of factors associated with hospitalizations due to mycoses, Brazil, 2000–2024.

| **Indicator/Variables** | **N (%)** | **Crude rate (per 100,000 inhabitants)** | **Adjusted rate (per 100,000 inhabitants) (95%CI)** | **RR (95%CI)** |
| --- | --- | --- | --- | --- |
| **Brazil – Total** | **47,376 (100.0)** | **0.97** | **0.96 (0.96-0.97)** | **-** |
| Chromoblastomycosis/ Chromomycosis | 651 (1.4) | 0.01 | 0.01 (0.01-0.01) | - |
| Coccidioidomycosis | 9,978 (21.1) | 0.20 | 0.20 (0.20-0.21) | - |
| Cryptococcosis | 11,308 (23.9) | 0.23 | 0.23 (0.23-0.23) | - |
| Histoplasmosis | 4,094 (8.6) | 0.08 | 0.08 (0.08-0.09) | - |
| Mycetoma | 710 (1.5) | 0.01 | 0.01 (0.01-0.02) | - |
| Paracoccidioidomycosis | 18,239 (38.5) | 0.37 | 0.37 (0.37-0.38) | - |
| Sporotrichosis | 2,435 (5.1) | 0.05 | 0.05 (0.05-0.05) | - |
| **Cause of hospital admission** |  |  |  |  |
| Primary | 42,973 (90.7) | - | - | - |
| Secondary | 4,403 (9.3) | - | - | - |
| **Death during hospital admission** |  |  |  |  |
| No | 42,905 (90.6) | - | - | - |
| Yes | 4,471 (9.4) | - | - | - |
| **HIV co-infection** |  |  |  |  |
| Yes | 2,586 (5.5) | - | - | - |
| No | 44,790 (94.5) | - | - | - |
| **Sex** |  |  |  |  |
| Female | 15,951 (33.7) | 0.64 | 0.64 (0.63-0.65) | *Reference* |
| Male | 31,425 (66.3) | 1.32 | 1.31 (1.29-1.32) | 2.05 (1.86-2.25) |
| *Missing data* | - | - |  |  |
| **Age group** |  |  |  |  |
| 0–14 | 7,652 (16.2) | 0.64 | 0.65 (0.63-0.66) | 1.22 (1.03-1.43) |
| 15–29 | 6,799 (14.4) | 0.52 | 0.54 (0.53-0.55) | *Reference* |
| 30–39 | 6,448 (13.6) | 0.86 | 0.84 (0.82-0.87) | 1.64 (1.39-1.95) |
| 40–49 | 7,922 (16.7) | 1.26 | 1.23 (1.21-1.26) | 2.41 (2.05-2.83) |
| 50–59 | 7,431 (15.7) | 1.59 | 1.52 (1.49-1.56) | 3.04 (2.58-3.59) |
| 60–69 | 5,330 (11.3) | 1.85 | 1.66 (1.62-1.71) | 3.54 (2.96-4.24) |
| ≥70 | 5,794 (12.2) | 2.47 | 2.23 (2.18-2.29) | 4.74 (3.98-5.65) |
| *Missing data* | - | - | - | - |
| **Ethnicityª** |  |  |  |  |
| Caucasian | 10,819 (22.8) | 0.48 | - | *Reference* |
| Afro-Brazilian / Afro-descendant | 1,392 (2.9) | 0.39 | - | 0.82 (0.62-1.08) |
| Asian-descendant | 414 (0.9) | 0.79 | - | 1.69 (1.04-2.74) |
| Mixed/ Pardo Brazilians | 11,437 (24.1) | 0.55 | - | 1.15 (1.01-1.32) |
| Indigenous (Amerindians) | 91 (0.2) | 0.44 | - | 1.02 (0.38-2.73) |
| *Missing data* | 23,223 (49,0) | - | - | - |
| **Region of residence** |  |  |  |  |
| North | 4,566 (9.6) | 1.12 | 1.28 (1.24-1.32) | 1.09 (0.90-1.31) |
| Northeast | 9,153 (19.3) | 0.68 | 0.69 (0.68-0.71) | 0.65 (0.56-0.76) |
| Southeast | 21,864 (46.1) | 1.06 | 1.03 (1.02-1.04) | 1.03 (0.90-1.18) |
| South | 7,217 (15.2) | 1.03 | 0.98 (0.96-1.01) | *Reference* |
| Central-West | 4,576 (9.7) | 1.28 | 1.27 (1.24-1.31) | 1.23 (1.02-1.48) |
| **Municipality size** |  |  |  |  |
| Small I | 9,100 (19.2) | 1.16 | 1.12 (1.10-1.15) | 1.27 (1.12-1.43) |
| Small II | 8,098 (17.1) | 1.03 | 1.02 (1.00-1.05) | 1.12 (0.99-1.27) |
| Medium | 5,349 (11.3) | 0.93 | 0.93 (0.91-0.96) | 1.02 (0.88-1.18) |
| Large | 24,829 (52.4) | 0.91 | 0.90 (0.89-0.92) | *Reference* |
| *Missing data* | - | - | - | - |
| **Residence in the capital** |  |  |  |  |
| No | 38,210 (80.7) | 1.03 | 1.02 (1.01-1.03) | 1.3 (1.16-1.46) |
| Yes | 9,166 (19.3) | 0.79 | 0.78 (0.77-0.80) | Ref |
| **IBP** |  |  |  |  |
| Very low | 9,704 (20.5) | 1.08 | 1.03 (1.01-1.05) | *Reference* |
| Low | 8,237 (17.4) | 0.92 | 0.90 (0.88-0.92) | 0.85 (0.73-0.98) |
| Medium | 9,864 (20.8) | 1.01 | 1.00 (0.98-1.02) | 0.93 (0.81-1.07) |
| High | 10,965 (23.1) | 1.13 | 1.14 (1.12-1.16) | 1.04 (0.91-1.19) |
| Very high | 8,604 (18.2) | 0.76 | 0.78 (0.76-0.80) | 0.70 (0.61-0.81) |
| *Missing data* | 2 (0.0) | - | - | - |
| **Typology of municipality** |  |  |  |  |
| Urban | 35,792 (75.5) | 0.97 | 0.96 (0.95-0.97) | *Reference* |
| Intermediate adjacent | 3,241 (6.8) | 1.02 | 1.01 (0.97-1.04) | 1.06 (0.88-1.26) |
| Intermediate remote | 383 (0.8) | 1.15 | 1.23 (1.10-1.35) | 1.16 (0.70-1.93) |
| Rural adjacent | 7,061 (14.9) | 0.96 | 0.95 (0.93-0.97) | 0.99 (0.88-1.13) |
| Rural remote | 897 (1.9) | 1.00 | 1.08 (1.01-1.16) | 1.03 (0.74-1.44) |
| *Missing data* | 2 (0.0) | - | - | - |

IBP: Brazilian Index of Deprivation (Índice Brasileiro de Privação); N: Number, %: Percentage; RR: Relative Risk; -: not calculated; ª ethnicity data available from 2008 for hospitalizations.
