# Supplementary material for: Neglected Mycoses in Brazil: A Population‐Based Study of Mortality and In‐Hospital Mortality Over 25 Years
Source: Mycoses. 2026 Feb 11;69(2):e70144. doi: 10.1111/myc.70144 (PMC12892236; doi:10.1111/myc.70144)
Supplement: Supplementary file 4 — Table S2: Temporal trends in crude hospitalisation rates for mycoses (per 100,000 inhabitants) by sociodemographic strata, Joinpoint regression, Brazil, 2000–2024. [file MYC-69-e70144-s002.docx]

**Supplementary material - Table 2:** Temporal trends in crude hospitalization rates for mycoses (per 100,000 inhabitants) by sociodemographic strata, Joinpoint regression, Brazil, 2000–2024.

| **Indicator/Variables** | **Period** | **APC (95%CI)** | **AAPC (95%CI)** |
| --- | --- | --- | --- |
| **Brazil – Total** | 2000−2008 | 5.54* (0.22;28.76) | -1.92* (-3.36;-0.56) |
|  | 2008−2024 | -4.38* (-8.96;-2.59) |  |
| **Sexª** |  |  |  |
| Female | 2000−2009 | 10.59* (3.98;34.11) | -1.86 (-4.13;0.37) |
|  | 2009−2024 | -6.72* (-12.27;-3.95) |  |
| Male | 2000−2002 | 44.09* (14.16;76.50) | -1.98* (-3.14;-0.90) |
|  | 2002−2024 | -2.71* (-3.68;-2.03) |  |
| **Age group** |  |  |  |
| 0–14 | 2000−2002 | 125.51* (14.67;327.26) | -5.51* (-9.20;-3.02) |
|  | 2002−2008 | 7.66 (-17.79;18.58) |  |
|  | 2008−2024 | -11.38* (-23.50;-5.86) |  |
| 15–29 | 2000−2024 | -2.02* (-3.39;-0.76) | -2.02* (-3.39;-0.76) |
| 30–39 | 2000−2024 | -2.96* (-4.16;-1.85) | -2.96* (-4.16;-1.85) |
| 40–49 | 2000−2024 | -2.66* (-3.60;-1.74) | -2.66* (-3.60;-1.74) |
| 50–59 | 2000−2010 | 2.76 (-0.48;12.10) | -2.52* (-3.70;-1.32) |
|  | 2010−2024 | -5.34* (-8.99;-3.57) |  |
| 60–69 | 2000−2016 | 1.65 (-0.66;30.21) | -1.53 (-3.25;0.43) |
|  | 2016−2024 | -9.03* (-31.94;-2.74) |  |
| ≥70 | 2000−2016 | 7.94* (3.59;19.33) | -1.58 (-5.73;3.39) |
|  | 2016−2024 | -24.40* (-44.97;-15.87) |  |
| **Ethnicityª** | − | − | − |
| Caucasian | − | − | − |
| Afro-Brazilian /Afro-descendant | − | − | − |
| Asian-descendant | − | − | − |
| Mixed/ Pardo Brazilians | − | − | − |
| Indigenous (Amerindians) | − | − | − |
| **Region of residence** | − | − | − |
| North | 2000−2009 | 16.56 (-3.42;43.19) | -3.68* (-7.04;-0.66) |
|  | 2009−2012 | -28.17 (-39.08;29.56) |  |
|  | 2012−2024 | -3.43 (-10.52;21.24) |  |
| Northeast | 2000−2016 | 6.50* (4.15;10.49) | 0.59 (-3.12;4.86) |
|  | 2016−2024 | -16.99* (-26.79;-11.76) |  |
| Southeast | 2000−2010 | 1.32 (-0.79;6.87) | -1.73* (-3.00;-0.55) |
|  | 2010−2013 | -16.99* (-22.33;-4.81) |  |
|  | 2013−2024 | 2.48* (0.19;7.99) |  |
| South | 2000−2002 | 140.38 (-1.30;400.89) | -2.63 (-6.08;0.50) |
|  | 2002−2016 | 0.55 (-32.38;17.63) |  |
|  | 2016−2024 | -16.79 (-51.32;24.72) |  |
| Central-West | 2000−2010 | 10.24 (-8.69;37.05) | -4.53* (-7.58;-1.93) |
|  | 2010−2013 | -27.96 (-38.17;25.45) |  |
|  | 2013−2024 | -4.72 (-12.30;27.29) |  |
| **Municipality size** |  |  |  |
| Small I | 2000−2009 | 10.89* (5.44;23.66) | -2.01 (-4.44;0.26) |
|  | 2009−2024 | -7.34* (-11.07;-5.11) |  |
| Small II | 2000−2008 | 6.23* (0.24;32.35) | -2.17* (-3.70;-0.74) |
|  | 2008−2024 | -5.00* (-10.14;-3.04) |  |
| Medium | 2000−2002 | 82.07* (19.80;137.24) | -3.46* (-5.25;-1.90) |
|  | 2002−2024 | -4.38* (-5.80;-3.35) |  |
| Large | 2000−2024 | -1.49* (-2.96;-0.07) | -1.49* (-2.96;-0.07) |
| **Residence in the capital** |  |  |  |
| No | 2000−2002 | 66.76* (12.98;126.05) | -2.11* (-3.65;-0.67) |
|  | 2002−2024 | -2.99* (-4.57;-1.97) |  |
| Yes | 2000−2009 | 5.17 (-1.03;47.10) | -1.39 (-3.20;0.38) |
|  | 2009−2024 | -4.26* (-21.74;-1.49) |  |
| **IBP** |  |  |  |
| Very low | 2000−2002 | 38.29 (-1.95;92.47) | -1.27 (-2.69;0.15) |
|  | 2002−2012 | -4.41 (-19.05;0.40) |  |
|  | 2012−2024 | 0.48 (-13.12;18.72) |  |
| Low | 2000−2024 | -2.42* (-3.50;-1.41) | -2.42* (-3.50;-1.41) |
| Medium | 2000−2010 | 8.01* (3.65;16.82) | -2.10* (-4.24;-0.08) |
|  | 2010−2013 | -22.35* (-29.96;-5.08) |  |
|  | 2013−2024 | -0.35 (-4.32;12.86) |  |
| High | 2000−2009 | 10.22* (2.25;39.57) | -2.56 (-5.33;0.01) |
|  | 2009−2024 | -7.94* (-15.73;-4.77) |  |
| Very high | 2000−2014 | 4.44* (1.76;8.86) | -1.23 (-4.02;1.49) |
|  | 2014−2024 | -11.03* (-18.34;-6.98) |  |
| **Typology of municipality** |  |  |  |
| Urban | 2000−2002 | 48.85* (1.70;96.51) | -2.02* (-3.37;-0.74) |
|  | 2002−2024 | -2.79* (-4.71;-1.80) |  |
| Intermediate adjacent | 2000−2009 | 13.72* (1.88;29.58) | -3.31* (-6.19;-0.78) |
|  | 2009−2012 | -26.06 (-35.59;19.20) |  |
|  | 2012−2024 | -3.26 (-9.00;21.58) |  |
| Intermediate remote | 2000−2024 | -0.13 (-2.93;3.05) | -0.13 (-2.93;3.05) |
| Rural adjacent | 2000−2014 | 5.83* (2.91;10.60) | -0.65 (-3.92;2.65) |
|  | 2014−2024 | -11.30* (-18.57;-7.12) |  |
| Rural remote | 2000−2024 | -3.06* (-5.48;-0.89) | -3.06* (-5.48;-0.89) |

*Significantly different from 0 (p < 0.05); 95% CI: 95% confidence intervals; AAPC: average annual percent change; APC: annual percent change; IBP: Brazilian Index of Deprivation (Índice Brasileiro de Privação); -: not calculated; ª ethnicity data available from 2008 for hospitalizations
