# Supplementary material for: Neglected Mycoses in Brazil: A Population‐Based Study of Mortality and In‐Hospital Mortality Over 25 Years
Source: Mycoses. 2026 Feb 11;69(2):e70144. doi: 10.1111/myc.70144 (PMC12892236; doi:10.1111/myc.70144)
Supplement: Supplementary file 5 — Table S3: HIV co‐infection in mycosis‐related: Mortality, Hospitalisations and Hospital mortality, Brazil, 2000–2024. [file MYC-69-e70144-s005.docx]

**Supplementary material - Table 3:** HIV co-infection in mycosis-related: Mortality, Hospitalizations and Hospital mortality, Brazil, 2000–2024.

|  | **Mortality** | | **Hospitalizations** | | **Hospital mortality** | |
| --- | --- | --- | --- | --- | --- | --- |
|  | **Total** | **HIV co-infection**  **N (%)** | **Total** | **HIV co-infection**  **N (%)** | **Total** | **HIV co-infection**  **N (%)** |
| **Mycoses** | **22,230** | **12,482 (56.1)** | **47,376** | **2,586 (5.5)** | **4,471** | **520 (11.6)** |
| Chromoblastomycosis/ Chromomycosis | 64 | 2 (3.1) | 651 | 2 (0.3) | 31 | 0 (0.0) |
| Coccidioidomycosis | 139 | 22 (15.8) | 9,978 | 18 (0.2) | 1,193 | 4 (0.3) |
| Cryptococcosis | 13,354 | 9,534 (71.4) | 11,308 | 1,713 (15.1) | 1,686 | 406 (24.1) |
| Histoplasmosis | 3,518 | 2,692 (76.5) | 4,094 | 474 (11.6) | 297 | 94 (31.6) |
| Mycetoma | 119 | 6 (5.0) | 710 | 0 0.0 () | 7 | 0 (0.0) |
| Paracoccidioidomycosis | 4,904 | 220 (4.5) | 18,239 | 138 (0.8) | 1,136 | 8 (0.7) |
| Sporotrichosis | 205 | 55 (26.8) | 2,435 | 247 (10.1) | 123 | 8 (6.5) |
